# Single-Neuron Adaptive Hysteresis Compensation of Piezoelectric Actuator Based on Hebb Learning Rules

**DOI:** 10.3390/mi11010084

**Published:** 2020-01-12

**Authors:** Yanding Qin, Heng Duan

**Affiliations:** College of Artificial Intelligence; Tianjin Key Laboratory of Intelligent Robotics, Nankai University, Tianjin 300350, China; 2120180411@mail.nankai.edu.cn

**Keywords:** piezoelectric actuator, hysteresis compensation, single-neuron adaptive control, Hebb learning rules, supervised learning

## Abstract

This paper presents an adaptive hysteresis compensation approach for a piezoelectric actuator (PEA) using single-neuron adaptive control. For a given desired trajectory, the control input to the PEA is dynamically adjusted by the error between the actual and desired trajectories using Hebb learning rules. A single neuron with self-learning and self-adaptive capabilities is a non-linear processing unit, which is ideal for time-variant systems. Based on the single-neuron control, the compensation of the PEA’s hysteresis can be regarded as a process of transmitting biological neuron information. Through the error information between the actual and desired trajectories, the control input is adjusted via the weight adjustment method of neuron learning. In addition, this paper also integrates the combination of Hebb learning rules and supervised learning as teacher signals, which can quickly respond to control signals. The weights of the single-neuron controller can be constantly adjusted online to improve the control performance of the system. Experimental results show that the proposed single-neuron adaptive hysteresis compensation method can track continuous and discontinuous trajectories well. The single-neuron adaptive controller has better adaptive and self-learning performance against the rate-dependence of the PEA’s hysteresis.

## 1. Introduction

As a sub-nanometer-resolution actuation device, piezoelectric actuators (PEAs) have been widely applied in various applications requiring nanometer-accurate motion [1,2,3,4]. However, the inherent hysteresis nonlinearity of the PEA greatly degrades its positioning accuracy, thus affecting its applicability and performance in precise operation tasks. The most significant characteristics of the PEA’s hysteresis are the rate-dependence and asymmetry [5,6,7], i.e., the hysteresis loop becomes thicker with the increment in the input rate (or frequency) and the hysteresis loop is not symmetric about the loop center. These characteristics increase the complexity of the system and cause great difficulties in hysteresis modeling and compensation.

To address the above problems, lots of control methods have been proposed to characterize and compensate the hysteresis of the PEA. Physical models can be derived from physical measurement methods, such as magnetization, stress–strain, and energy principles [8,9]. However, the mathematical representations are often complex, making it difficult to obtain the inverse hysteresis model. In the meantime, a phenomenon-based model is also proposed, such as the Preisach model [10], Prandtl–Ishlinskii (PI) model [11,12], and Maxwell model [13]. As the inversion of the classical PI model is analytically available, it has been widely utilized in much research to describe the hysteresis characteristics of the PEA. After the inversion model is obtained, it can be utilized as a feedforward hysteresis compensator. This modeling and inversion approach is widely adopted, and many adaptive methods can be integrated [14,15,16,17]. In order to avoid the inversion calculation, the direct inversion method (DIM) is also proposed to identify the inverse hysteresis model directly from the measurements in parameter identification [18,19,20].

For model-based hysteresis compensation, the performance of the controller is highly dependent on the modeling accuracy of the hysteresis model. However, the PEA’s hysteresis is susceptible to many factors, such as the external load and the frequency of the control input. This makes the modeling and compensation of the PEA’s hysteresis very case-sensitive. As a result, a high-precision hysteresis model is generally difficult to obtain. Therefore, many intelligent control algorithms have been proposed to achieve higher robustness and adaptability. For instance, sliding mode control has been proposed to improve the accuracy and the robustness against noise and disturbances [14,21]. A linearization control method with feedforward hysteresis compensation and proportional-integral-derivative (PID) feedback has also been proposed [22]. Besides, iterative learning control schemes have been verified to achieve high-performance tracking for PEAs [23].

In the field of intelligent control, the neural network is a highly powerful system identification tool. It has a strong self-learning ability and powerful mapping ability to nonlinear systems, which has been widely used in the control of complex systems [24,25]. In the hysteresis compensation of the PEA, Wang and Chen presented a novel Duhem model based on the neural network to describe the dynamic hysteresis of PEAs [26]. An inversion-free predictive controller was proposed based on a dynamic linearized multilayer feedforward neural network model [27]. A cerebellar model articulation controller neural network PID controller was also proposed [4]. A radial basis function (RBF) network was also used to model and compensate for the PEA’s hysteresis [28]. However, the use of the S-type action function increases the calculation difficulty for fast, high-frequency, and fast-response systems such as the PEA.

Among the neural network-based controllers, the single adaptive neuron system retains the advantages of the neural network and can satisfy the requirements of the real-time control of fast processes [29,30]. Therefore, a single-neuron adaptive hysteresis compensation method is proposed in this paper. The controller imitates an adaptive single-neuron system to learn and uses Hebb learning rules and supervised learning to adjust the controller. The controller can respond quickly to time-varying signals, making it suitable for the rate-dependent hysteresis compensation. Positioning and trajectory tracking experiments are carried out to investigate the performance of the proposed method. The performance of the PID control is also investigated for the purpose of comparison. For the positioning control, the proposed method can converge in about 8 ms and the steady-state tracking error can be reduced to the noise level of the system. For trajectory tracking, sinusoidal and triangular trajectories with frequencies up to 50 Hz are utilized. The experimental results show that the proposed method has excellent robustness and adaptability against the rate-dependence of the PEA’s hysteresis, and the hysteresis can be successfully compensated.

This paper is organized as follows: Section 2 introduces the properties of the inherent hysteresis of the PEA. Section 3 presents the single-neuron adaptive controller design and analysis. To investigate the efficiency of the proposed method, experimental verifications and performance analyses are provided in Section 4. Section 5 summarizes this paper.

## 2. The Hysteretic Nonlinearity of the PEA

### 2.1. Experimental Setup

**Figure 1:**
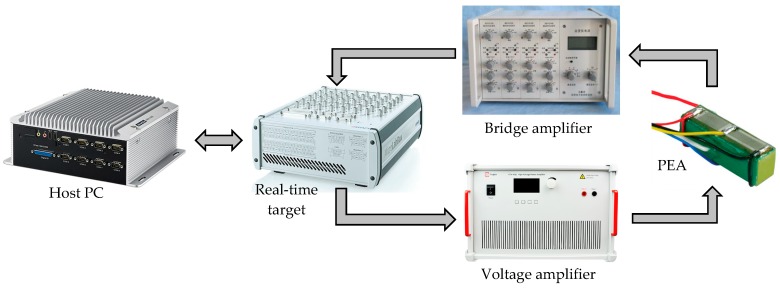
Schematic of the system setup for the standalone piezoelectric actuator (PEA).

As shown in Figure 1, in this paper, a standalone PEA (model PZS001 from Thorlabs with integrated strain gauge sensors, Newton, NJ, USA) with a high-voltage amplifier (model ATA-4052 from Aigtek with a bandwidth of DC-500 kHz, Xi’an, China) is selected as the plant. The maximum displacement output of the PEA is measured to be 12.925 μm under the maximum actuation voltage of 10 V, i.e., the actuation gain (displacement/voltage) is 1.2925. According to the datasheet, the resonant frequency of the PEA used in this paper is 69 kHz. A dynamic Wheatstone bridge amplifier (model SDY2105 from Beidaihe Institute of Practicality Electron Technology with a bandwidth of DC-300 kHz, Qinhuangdao, China) is utilized to measure the strain of the PEA, which is used to calculate the displacement of the PEA. The data acquisition and closed-loop control tasks are implemented on a real-time target (model microlabbox from dSPACE, Paderborn, Germany) with a sampling rate of 10 kHz. The algorithm is programmed in Simulink and implemented in Controldesk. Due to the influence of the strain gauges and the Wheatstone bridge amplifier, the measurement noise of the overall system is found to be ±34 nm.

### 2.2. Characteristics of the PEA’s Hysteresis

**Figure 2:**
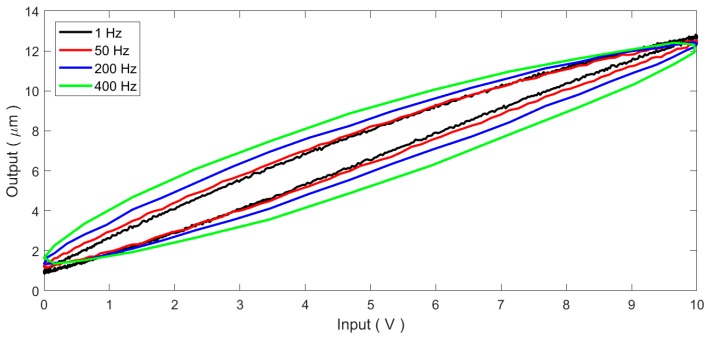
The measured hysteresis loops of the standalone PEA.

Obvious nonlinearities can be observed in the input–output relationship of the PEA. Generally, the hysteresis is the dominant factor affecting the motion accuracy of the PEA. This paper uses sinusoidal signals of *u*(*t*) = 5sin(2π*ft* − π/2) + 5 to drive the PEA at different frequencies. By observing the input signal and the measured displacement output of the PEA, hysteresis loops of the PEA can be obtained. Figure 2 depicts the measured input–output loops of the standalone PEA. As the resonant frequency of the PEA is 69 kHz, within the driving frequency of 1–400 Hz, the dynamics of the PEA can be neglected. As a result, the measured input–output loops shown in Figure 2 are totally produced by the hysteretic nonlinearity of the PEA. It can be seen that there is an obvious rate-dependent behavior in the measured hysteresis loops. As the input frequency increases, the hysteresis loop grows bigger and thicker. In addition, the hysteresis loop is not strictly symmetric about the loop center. The above rate-dependence and asymmetry properties increase the model complexity and increase the difficulty in the controller design of the PEA. Therefore, how to compensate the hysteresis and linearize the system have become crucial problems of the PEA.

## 3. Single-Neuron Adaptive Controller Design

### 3.1. Single-Neuron Adaptive Control Algorithm

Aiming to compensate the hysteresis of the PEA, this paper proposes a single-neuron adaptive controller without modelling the hysteresis of the PEA. A single neuron is a non-linear processing unit that has self-learning and self-adaptive capabilities and is applicable for many different control tasks. The input and output of a single-neuron system are expressed as follows:

(1) $$y=K\cdot \sum_{i=1}^{n}w_{i}x_{i}+\delta ,$$

where *K* denotes the gain characterizing the response speed of a neuron; *x_i_*, *y*, and *δ* are the state variable, output, and threshold, respectively; and *w_i_* represents the weight of *x_i_* that can be adjusted by the learning rules.

Neurons are generally considered to be self-organizing by modifying their synaptic weighting values. Supervised Hebb learning rules are usually used for the adjustment of the weights. Assuming the weight of the neuron *w_i_*(*t*) during learning is proportional to the signal *p_i_*(*t*) and decays slowly, the learning rule of the neuron can be expressed as

(2) $$w_{i}\left(t+1\right)=\left(1-c\right)w_{i}\left(t\right)+dp_{i}\left(t\right),$$

where *c* is a positive constant that determines the impact of the last weight value, *d* is a constant characterizing the learning efficiency, and *p_i_*(*t*) is the learning rules. To further improve the adaptability of neurons, the following learning rules are employed:

(3) $$p_{i}\left(t\right)=Z\left(t\right)S\left(t\right)x_{i}\left(t\right),$$

where *S*(*t*) indicates that the adaptive neuron adopts the Hebb learning rule, and *Z*(*t*) shows supervised learning rules. *Z*(*t*) means that external information is self-organized to have a control effect under the guidance of the teacher signal. In this way, the adaptive neuron algorithm combined with Hebb learning rules and supervised learning can perform self-organizing and adaptive control for nonlinear systems.

### 3.2. Controller Design for the PEA

**Figure 3:**
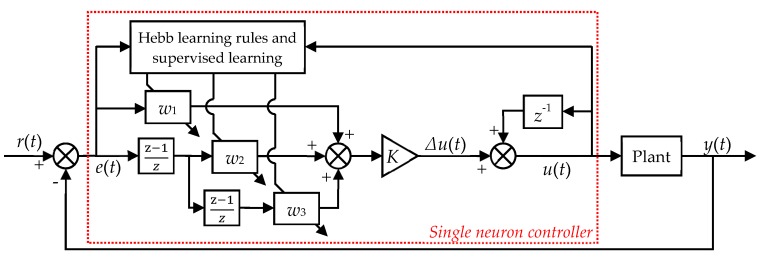
Schematic diagram of the single-neuron adaptive hysteresis compensation method.

As shown in Figure 3, the state variables to the controller are calculated by the error between the desired trajectory *r*(*t*) and the actual trajectory *y*(*t*). The output of the controller is *u*(*t*). In order to ensure the convergence and robustness of the learning algorithm, the following modified adaptive learning algorithm is adopted in this paper:

(4) $$\begin{array}{l} x_{1}\left(t\right)=e\left(t\right)\\ x_{2}\left(t\right)=\Delta x_{1}(t)=e\left(t\right)-e\left(t-1\right)\\ x_{3}\left(t\right)=\Delta x_{2}(t)=e\left(t\right)-2e\left(t-1\right)+e\left(t-2\right) \end{array},$$

where *e*(*t*) = *r*(*t*) − *y*(*t*) is the error between the desired and actual trajectories, and *x*_1_(*t*), *x*_2_(*t*), and *x*_3_(*t*) are adopted as the state variables to the neuron system.

The previous controller output *u*(*t*−1) can be utilized as the threshold, i.e., *δ* = *u*(*t* − 1). Substituting this into Equation (1), the controller output of the single-neuron adaptive controller can be written as follows:

(5) $$u\left(t\right)=K\cdot \sum_{i=1}^{n}w_{i}x_{i}+u\left(t-1\right),$$

For the adaption of the weights, *Z*(*t*) = *e*(*t*) is adopted as the supervisory function and *S*(*t*) = *u*(*t*) is adopted as the Hebb learning rule. Substituting these into Equations (2) and (3), the learning rule of the neuron can be expressed as follows:

(6) $$w_{i}\left(t\right)=w_{i}\left(t-1\right)+d\cdot e\left(t\right)\cdot u\left(t\right)\cdot x_{i}\left(t\right),$$

where *c* is set as 0 because *w_i_*(*t*) will converge to a stable value if *c* is small enough. According to the common experience of single-neuron adaptive control, *d* is typically less than 0.5. In this paper, *d* = 0.4 is adopted.

The whole control progress proceeds as follows. After getting the desired trajectory and actual trajectory, the state variable *x*_i_(*t*) is calculated using Equation (4). Three state variables correspond to three control outputs produced by the neuron, which are the proportional feedback *u*_1_(*t*), first-order differential feedback *u*_2_(*t*), and second-order differential feedback *u*_3_(*t*), respectively. The proportional feedback can quickly reduce the tracking error. The first-order differential feedback can improve the system’s transient state performance, i.e., the response speed and overshoot. The second-order differential feedback ensures that the system remains stable during a fast response. The change in the weight reflects the dynamic characteristics of the controlled plant and the response process. The neuron continuously adjusts the weight through its own learning rules, and quickly eliminates the error and enters the steady-state under the correlation of the three feedbacks.

The system response speed is positively proportional to *K*, but a large overshoot might make the system unstable. On the contrary, if *K* is too small, the actual trajectory cannot track the desired trajectory. Thus, the tuning of *K* is very important. In order to determine the proper value for *K*, we built a mathematical model for the PEA using the Prandtl–Ishlinskii model. Through several simulation tests, the influence of *K* is computationally investigated. A candidate *K* is then selected according to the simulation results. Subsequently, this candidate *K* is adopted as the initial value and it is tuned manually online to achieve improved tracking performance. In this case, only fine tuning within a very small range is necessary.

## 4. Experimental Verifications

On the basis of the above analyses, the single-neuron adaptive control algorithm is applied to compensate the PEA’s hysteresis. Positioning and trajectory tracking experiments are carried out to verify the proposed method’s performance in hysteresis compensation.

In order to better compare the performances, this paper also includes the open loop and PID control results for the purpose of comparison. For the open-loop control, the PEA is assumed to be linear and the actuation gain (the ratio between the maximum allowable control input and the maximum displacement output) is utilized to finish the input–output mapping, i.e.,

(7) $$u(t)=r(t)\cdot \frac{U_{\max}}{Y_{\max}},$$

where *U*_max_ and *Y*_max_ are the maximum allowable control input and maximum displacement output, respectively. The open-loop control represents the basic characteristics of the system as no controller is utilized.

PID control, a widely utilized controller, has the advantages of simple parameter adjustment and ease of use. However, for nonlinear systems such as the PEA, the tuning of the gains in the PID controller is not an easy task. It might not work properly to systematically adjust the PID gains via strictly following well-developed approaches such as the Ziegler–Nichols method. Further, the behavior of the PEA is susceptible to many factors, making it difficult or impossible for the PID control to maintain the control performance in all scenarios. All these increase the difficulty in PID tuning. In this paper, the critical ratio method is adopted to tune the PID gains. At the beginning, only the proportional gain *K_p_* is tuned with the other gains set to 0. Subsequently, the other gains are adjusted after the *K_p_* is specified. For the PEA, PI control is found to be adequate to achieve satisfactory performance. In fact, a trial and error process is inevitable to finely tune the PID gains to achieve satisfactory results.

### 4.1. Step Response

**Figure 4:**
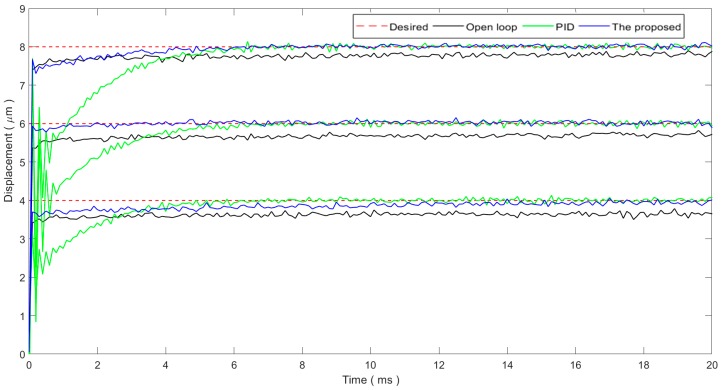
Step response performance.

The step response is generally utilized to test the system’s positioning accuracy. Further, it can also show the system’s tracking performance for non-continuous trajectories. For step response, the gains of the proposed single-neuron adaptive controller are tuned to be *K* = 0.0002 and *d* = 0.4 following the method provided in Section 3.2. The gains of the PID controller are manually tuned to be *K_p_* = 0.8, *K_i_* = 1000, and K*_d_* = 0 following the critical ratio method. Step response experiments are carried out and the experimental results are shown in Figure 4. The step response of the open-loop system is also provided for the purpose of comparison.

As the resonant frequency of the PEA is 69 kHz and the sampling rate is set to 10 kHz, the transient state of the open-loop system is not observable. However, the slow creeping of the PEA can be observed and the steady-state positioning error is quite large. As a result, for the PEA, the response is fast, whereas the steady-state positioning accuracy is low. For the PID control, the PEA can converge within 8 ms and the steady-state positioning error is reduced to the noise level, whereas oscillations can be found in the transient state. For the proposed single-neuron adaptive controller, the rise time is on the same level of the open-loop system, indicating a fast response. There are no oscillations in the transient state. The proposed controller can also reduce the steady-state positioning error to the noise level. The convergence time, i.e., the learning time, of the proposed controller is on the level of several milliseconds. The experimental results show that the learning time of the proposed controller has an obvious sensitivity to the magnitude of the step size. For a smaller magnitude step, e.g., 4 μm step, the proposed controller can respond quickly, whereas it will take a relatively longer time (approximately 14 ms) for the controller to converge. For larger magnitudes, e.g., 8 μm step, the proposed controller can respond quickly and converge in about 6 ms.

Based on the above experimental results, both the proposed controller and the PID controller can reduce the steady-state positioning error to the noise level. The proposed controller is superior to the conventional PID controller in that it achieves a fast response and smooth transient state behavior. For the convergence time, the proposed controller converges faster for large step values, whereas the convergence time increases for small step values.

### 4.2. Tracking of Sinusoidal Trajectories

Sinusoidal trajectories are selected in this paper to verify the tracking performance of the proposed method on continuous trajectories. First, a low-frequency trajectory is adopted to tune the parameters of the controllers. In this case, the rate-dependence of the PEA’s hysteresis can be neglected. This helps to ease the tuning of the parameters. The parameters of the controllers are tuned until excellent tracking performance is achieved in this case. These values are then fixed and higher-frequency trajectories are utilized to test the robustness and adaptability of the controllers.

**Figure 5:**
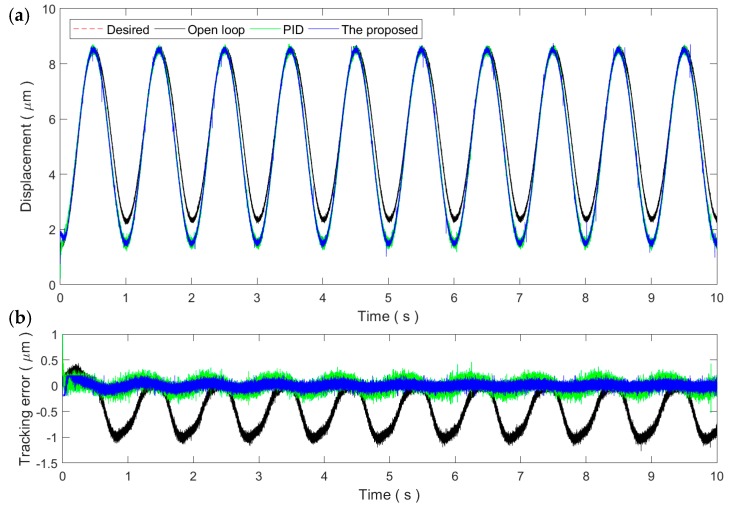
Tracking of the 1 Hz sinusoidal trajectory: (**a**) Time plot and (**b**) tracking error.

In this paper, a 1 Hz sinusoidal trajectory is utilized to tune the parameters of the proposed method and the PID controller. For the proposed method, the parameters are tuned to *K* = 0.002 and *d* = 0.4. For the PID controller, the gains of *K_p_*, *K_i_*, and K*_d_* are tuned to 1.11, 100, and 0, respectively. Figure 5 shows the tracking performance of the 1 Hz sinusoidal trajectory. Compared to the open-loop system, both the proposed method and the PID controller can successfully compensate the hysteresis of the PEA. The PEA can follow the desired trajectory well. It can be observed that the steady-state tracking error of the proposed method can be reduced to the noise level. The steady-state tracking error of the PID controller is slightly higher than the noise level but is still comparable to the proposed method. In the following experiments, sinusoidal and triangular trajectories with higher frequencies are utilized while the parameters of the two controllers are fixed to the above values. This helps to test the robustness and adaptability of the proposed method against the rate-dependence of the PEA’s hysteresis.

**Figure 6:**
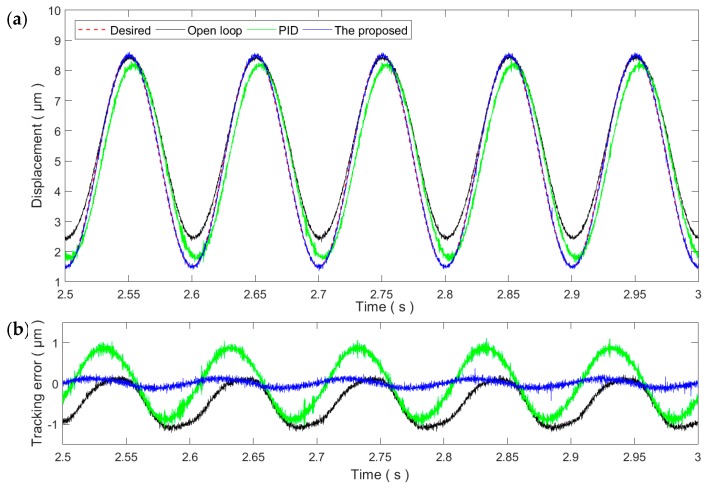
Tracking of the 10 Hz sinusoidal trajectory: (**a**) Time plot and (**b**) tracking error.

**Figure 7:**
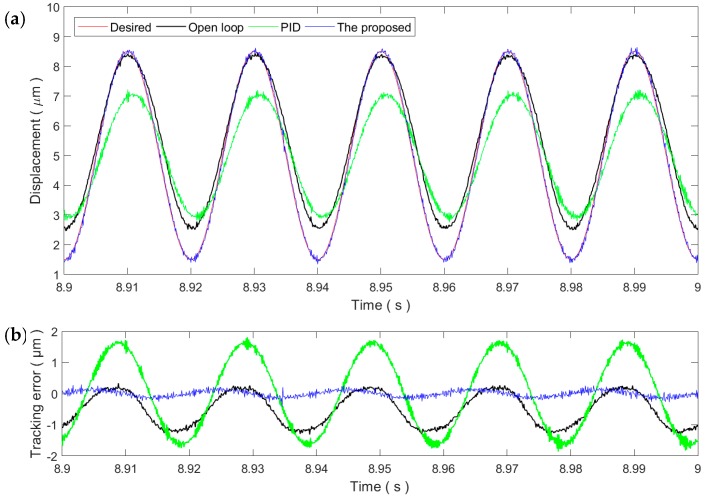
Tracking of the 50 Hz sinusoidal trajectory: (**a**) Time plot and (**b**) tracking error.

Figure 6 and Figure 7 show the sinusoidal trajectory tracking results at 10 and 50 Hz, respectively. As the frequency of the desired trajectory increases, the trajectory tracking error of the proposed method increases slightly but still remains at the same magnitude of the measurement noise, exhibiting high robustness and adaptability against the rate-dependence of the PEA’s hysteresis. On the contrary, the tracking performance of the PID controller starts to drop significantly at the frequency of 10 Hz. For the 50 Hz trajectory, the tracking performance of the PID control is even lower than the open-loop control. As a result, the PID gains tuned at the 1 Hz trajectory is only applicable for slow trajectories and might not work properly for fast trajectories.

More trajectory tracking experiments are performed, whereas not all the experimental results are presented for the conciseness of this paper. In order to quantitatively investigate the tracking performance, the root-mean-square tracking error (RMSE) and relative root-mean-square error (RRMSE) of these sinusoidal trajectories are calculated and presented in Table 1. RMSE and RRMSE are defined in the following equations:

(8) $$RMSE=\sqrt{\sum_{i=1}^{N}{\left(y_{i}-r_{i}\right)}^{2}/N},$$

(9) $$RRMSE=\sqrt{\sum_{i=1}^{N}{\left(y_{i}-r_{i}\right)}^{2}/\sum_{i=1}^{N}r_{i}}\times 100\%,$$

where *y_i_* and *r_i_* represents the *i* th values of the actual and desired trajectories, respectively, and *N* is the length of sampling data.

As the parameters of the controllers are tuned at the 1 Hz trajectory, the RMSEs of the proposed method at the PID control are 64.5 and 101.9 nm, corresponding to a 0.76% and 1.2% relative error, respectively. As the frequency increases, the tracking performances of both controllers start to degrade. For the proposed method, the RMSE increases to 170.2 nm, i.e., 2.02% relative error. On the contrary, the RMSE of the PID control increases to 1158.8 nm, an approximately 13.63% relative error. These experimental results demonstrate that the proposed method can successfully compensate the rate-dependent hysteresis of the PEA.

### 4.3. Tracking of Triangular Trajectories

Sinusoidal trajectories are smooth trajectories that do not contain high-frequency harmonic components. In applications, triangular trajectories are also widely utilized, e.g., the scanning of the sample in an atomic force microscope. Unlike sinusoidal trajectories, triangular trajectories contain high-frequency harmonic components, increasing the difficulty in control. Therefore, the triangular trajectory is also selected in this paper to verify the performance of the proposed method for non-smooth trajectories. The parameters of the PID controller and the proposed method obtained in Section 4.2 are also inherited.

**Figure 8:**
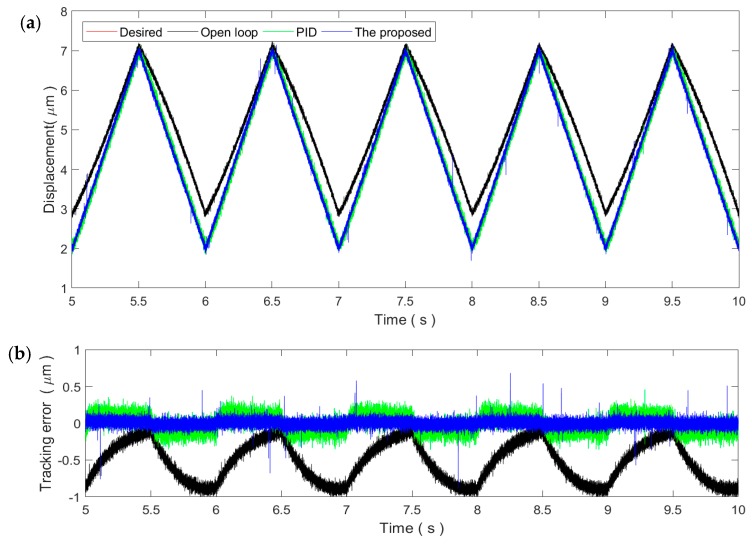
Tracking of the 1 Hz triangular trajectory: (**a**) Time plot and (**b**) tracking error.

**Figure 9:**
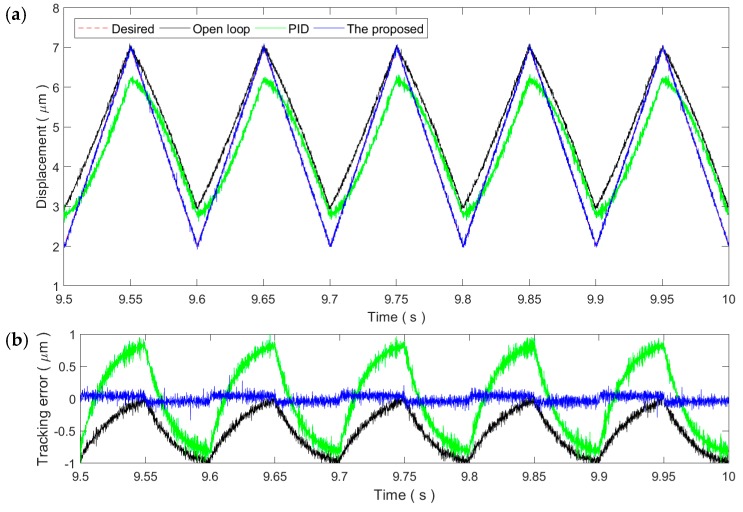
Tracking of the 10 Hz triangular trajectory: (**a**) Time plot and (**b**) tracking error.

**Figure 10:**
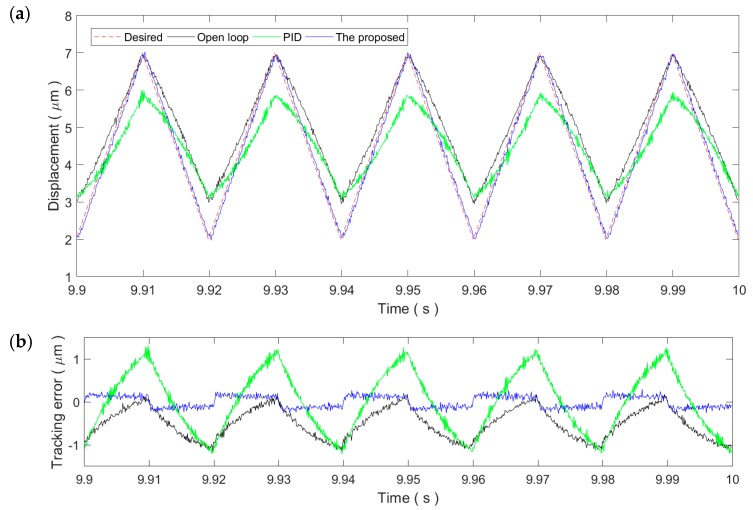
Tracking of the 50 Hz triangular trajectory: (**a**) Time plot and (**b**) tracking error.

As shown in Figure 8, for the 1 Hz triangular trajectory, the tracking performance is similar to the 1 Hz sinusoidal trajectory. Both the PID control and the proposed method can reduce the tracking error to the noise level. This demonstrates the applicability of the two controllers for slow trajectories. Similarly, the tracking performance of both controllers decreases with the increment in the frequency of the triangular trajectory, which is obvious in Figure 9 and Figure 10. Due to the influence of the high-frequency harmonic components, the tracking performance of the proposed method on the triangular trajectories is slightly lower than that of the sinusoidal trajectories. However, the tracking error still remains at very small ranges, showing strong robustness against the rate-dependence.

### 4.4. Hysteresis Compensation Efficiency

**Figure 11:**
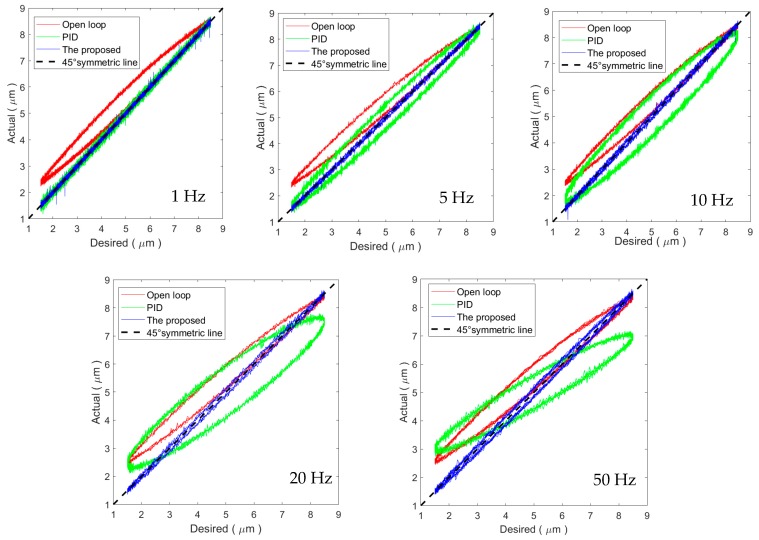
Hysteresis compensation efficiency for the sinusoidal trajectories.

The trajectory tracking results for sinusoidal trajectories are utilized to analyze the hysteresis compensation efficiency of the proposed method. The hysteresis loops in these experimental results are shown in Figure 11, where a 45° line is included to show the unitary mapping from the desired trajectory to the actual trajectory. For the 1 Hz sinusoidal trajectory, the resultant input–output relationship of both the PID controller and the proposed method coincide with the 45° line. The hysteresis loop is not observable, which means the hysteresis has been efficiently compensated. As the frequency increases, the input–output relationship of the proposed method stays close to the 45° line. On the contrary, for the PID controller, obvious hysteresis loops can still be observed in the input–output relationship for sinusoidal trajectories higher than 5 Hz. From Figure 11, we can conclude that the proposed method can successfully compensate the rate-dependent hysteresis of the PEA when compared to the PID controller.

## 5. Conclusions

The rate-dependence and asymmetry of the PEA’s hysteresis increase the difficulty in the hysteresis modeling and compensation. Further, the PEA’s hysteresis is susceptible to the system’s configurations, making the hysteresis compensation of PEAs very case-sensitive. In this paper, a single-neuron adaptive hysteresis compensation method is proposed. The supervised learning and Hebb learning rules are adopted to dynamically adjust the weights of the neurons according to the error between the actual and desired trajectories and their first-order and second-order differences. As a branch of neural network control, the single-neuron adaptive control simplifies the training process of neural network control while retaining the advantages of neural network control. The learning efficiency and convergence are improved. Positioning control results show that the proposed method can reduce the steady-state tracking error to the noise level, and the transient state performance can be guaranteed. The experimental results of tracking sinusoidal and triangular trajectories with frequencies up to 50 Hz show that the proposed method can successfully compensate the rate-dependent hysteresis of the PEA. The steady-state tracking error can be maintained in a small range, showing great robustness and adaptability against the rate-dependence. Future work will focus on further improving the tracking performance for higher-frequency trajectories.

## Figures and Tables

**Table 1:** Tracking errors of proportional-integral-derivative (PID) and the proposed approach.

Frequency (Hz)	The Proposed Method		PID	
	RMSE (nm)	RRMSE (%)	RMSE (nm)	RRMSE (%)
1	64.5	0.76	101.9	1.20
5	90.2	1.06	349.3	4.11
10	108.6	1.28	626.1	7.37
20	133.0	1.56	941.5	11.08
50	170.2	2.02	1158.8	13.63
